# Strongyloidiasis in northern Vietnam: epidemiology, clinical characteristics and molecular diagnosis of the causal agent

**DOI:** 10.1186/s13071-019-3776-1

**Published:** 2019-11-04

**Authors:** Nguyen Van De, Pham Ngoc Minh, Le Van Duyet, Santiago Mas-Coma

**Affiliations:** 1 Hanoi Medical University, 01 Ton That Tung, Hanoi, Vietnam; 2 National Hospital of Tropical Diseases 78 Giai Phong, Hanoi, Vietnam; 3 Departamento de Parasitología, Facultad de Farmacia, Universidad de Valencia, Av. Vicent Andrés Estellés s/n, Burjassot, 46100 Valencia, Spain

**Keywords:** *Strongyloides stercoralis*, Epidemiology, Geographical distribution, Symptomatology, Diagnosis, ITS1 sequence, Vietnam

## Abstract

**Background:**

Strongyloidiasis is a health problem in Vietnam, but appropriate information is still limited. The aim of this study was to determine the prevalence, geographical distribution, epidemiological aspects, symptoms and other health indicators of *Strongyloides stercoralis* infections in patients from 27 provinces of northern Vietnam attending the Hanoi Medical University Hospital during 2016 and 2017.

**Methods:**

Blood samples of 2000 patients were analyzed for *S. stercoralis* infection with an IgG ELISA test. Seroprevalence was analyzed by gender, age group, locality of origin (rural or urban areas) and symptoms. Stools from the seropositive patients were examined for the detection of worms which were subsequently used for species identification by morphology and rDNA ITS1 sequencing.

**Results:**

A seroprevalence of 20% was detected, showing an increasing prevalence from young to older age groups but without significant gender difference. Seroprevalence was higher in rural areas than in urban areas, both in general and individually in all provinces without exception, and lower in the mountainous areas than in the large valley lowlands. The follow-up of the 400 patients showed eosinophilia in 100% of cases, diarrhoea in 64.5%, digestion difficulties in 58.0%, stomachache in 45.5%, stomach and duodenal ulcers in 44.5%, itching in 28.0% and fever in 9.5%. The prevalence of symptoms and signs were also higher in older age groups than in younger age groups. Worms were detected in stools of 10.5% of the patients. Sequencing of a 501-bp nuclear ribosomal DNA ITS1 fragment allowed for the verification of infection by *Strongyloides stercoralis*.

**Conclusions:**

To our knowledge, this study is the largest survey of human strongyloidiasis in Vietnam so far and the first molecular identification of this nematode species in this country. Long-term chronicity may probably be usual in infected subjects, mainly in the older age groups.

## Background

Strongyloidiasis is a helminthic disease included among the group of soil-transmitted diseases (STDs), although still not included within the neglected tropical diseases list (NTDs) [1]. It is caused by small-sized, soil-transmitted nematodes whose females and larval stages infect humans, dogs and cats [2–4]. Their life-cycle includes a generation of adult males and females free-living in humid soil and a parasitic parthenogenetic female generation infecting the host’s intestine. Human infection takes place by transcutaneous or transmucosal penetration of strongyloid filariform larvae issued from rhabditiform larvae in the soil under appropriate conditions. After penetration, infective larvae migrate through the blood system, proceeding to the lungs *via* the heart and from the alveoli to the trachea and subsequent swallowing into the oesophagus, stomach and small intestine, mainly the duodenum, where adult females develop and produce eggs by parthenogenesis. Rhabditiform larvae hatch from these eggs in the intestine and are expelled with the faeces [5].

*Strongyloides stercoralis* may cause dermatitis at the site of invasion, lesions in the lungs and bronchitis due to the migrating larval stage [6]. The main lesions in strongyloidiasis are seen in the digestive tract, especially the duodenum and the upper part of the jejunum, but may occur also in the bile and pancreatic ducts. Strongyloidiasis may cause intermittent symptoms that mostly affect the intestine (abdominal pain and intermittent or persistent diarrhoea), the lungs (cough, wheezing, chronic bronchitis) or skin (pruritus, urticaria) [1]. Once autoinfection starts, additional lesions caused by the larvae aggravate the mucosal damage such as erosions and ulcers, with the possible destruction of the muscular layer which may lead to perforation. Symptoms include abdominal discomfort, right upper abdominal pain, diarrhoea, irregular fever and cough. These symptoms become aggravated in autoinfection including mucous, bloody diarrhoea, anaemia, edema and ascites [7].

Clinical complications in organs other than the duodenum have also been described [8–10]. The health effects of strongyloidiasis on pregnant women should also be considered [11]. Autoinfection of the patients may lead to highly problematic, severe hyperinfections which are almost invariably fatal [12–18].

Strongyloidiasis is also known as one of the few helminthiases linked to immunosuppression situations, such as in AIDS [19, 20], organ transplantation post-surgery [21] or other processes [22, 23]. This intestinal disease may secondarily give rise to the so-called larva currens, dermic lesions remembering cutaneous larva migrans by hookworms but differentiated from the latter by the linear courses of the skin lesions, their higher movement speed, their appearance mostly at the level of the back or abdomen, lower eritema and the absence of secondary bacterial infection after scratching [24].

For diagnosis of patients, coprology for rhabditiform larvae detection in the patient’s stools has been noted to be of low sensitivity, and serology has been therefore recommended as diagnostic method [25–27].

Strongyloidiasis is widely distributed, with an estimated 30 to 100 million infected individuals throughout the world [27], especially in the tropical regions characterized by high temperatures and humidity and poor hygienic conditions. In Africa, the range of infection prevalences in the communities varies from 0.1% in the Central African Republic up to 91.8% in Gabon. In the Gisagara District, Southern Province, Rwanda, *S. stercoralis* infection was found to be 17.4% [28]. In South and Central America, Haiti reports a prevalence of 1.0%, while in Peru the infection prevalence is as high as 75.3% [3].

In Southeast Asia, another highly endemic part of the world, several countries reported infection prevalences within a comparably small range [29–31]. In Cambodia the infection prevalence was 17.5%, in Thailand 23.7% and in Lao PDR 26.2% [3].

In Vietnam, there are already reports of human infection [32–36]. However, studies on strongyloidiasis are limited. Using stool examinations, the results in communities showed a prevalence of 0.2–2.5% [6]. When sero-immunological tests were used, the prevalence detected was higher, such as strongyloidiasis infection prevalences of 29% in the stomachache patient group and 7.6% at the community level [37], and 7.6% in other communities [33]. In recent years, many thousands of strongyloidiasis infections have been detected in hospitals. However, an overview about the strongyloidiasis situation in Vietnam, particularly with a wider evaluation by analyzing a larger number of subjects, a study to assess *Strongyloides stercoralis* infection in the northern part of the country, including the capital Hanoi and surroundings, and a characterization of the causal agent species by a molecular method, is still needed.

## Methods

### Sample collection

A total of 2000 adult patients (> 15 years-old) visited the Hanoi Medical University Hospital from 2016 to 2017 for examination. The Hanoi Medical University Hospital receives patients from throughout the whole country, although only patients from northern Vietnam were considered for this survey. The present study only included patients presenting with clear symptoms sent by polyclinical doctors. The *Strongyloides stercoralis* test was applied as a routine diagnosis by the Parasitology Department of the hospital.

**Fig. 1:**
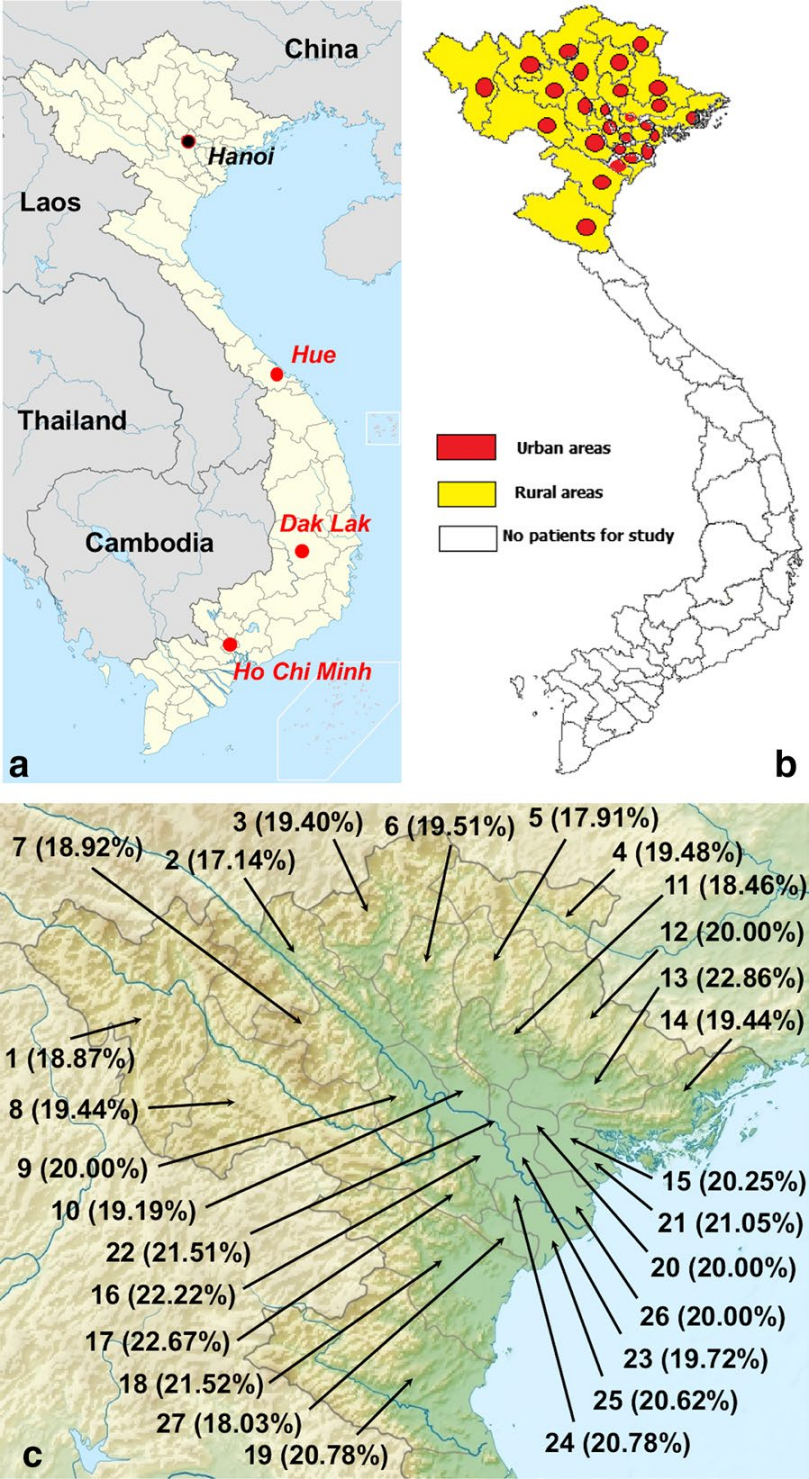
Maps of Vietnam showing political division in provinces. **a** Location of Vietnam in Southeast Asia, showing areas where human strongyloidiasis has been previously analyzed in the country: Ho Chi Minh city (seroprevalence of 18.2%), Dak Lak in rural central highlands (42.4%), Hue in the central coast area (29.9%) and Hanoi city where the Medical University Hospital receiving the patients studied is located. **b** Distribution of urban and rural areas of northern Vietnam where the patients live. **c** Geographical map of northern Vietnam showing seroprevalences (in parentheses) according to the 27 provinces analyzed (for province names see corresponding numbers in Table 2)

Information collected consisted of gender (male/female), locality of origin (from rural or urban areas in each province) (Fig. 1), age group (16–25; 26–35; 36–45; 46–55; 56–65; and > 65 years-old), data of serum collection (*S. stercoralis* IgG ELISA tests), blood characteristics and clinical symptoms. The following patients were chosen: those who presented with eosinophilia (> 8%, according to the normal physiology of Vietnamese inhabitants) and positive ELISA test using *S. stercoralis* antigen (IgG ELISA kit of a DRG Instruments GmbH, Springfield, USA) with a sensitivity of 100% and a specificity of 98%. Parasitic samples included 85 worms in total collected from patients and kept in 70% ethanol at − 20 °C for the molecular identification of the causal agent species. The present study did only focus on strongyloidiasis, stool samples from the patients being examined the day of collection with microscopic preparations of stool smears.

### Clinical analyses

Main symptoms considered included diarrhoea, digestive disorders, stomachache, itching, fever and bloody stool. Additionally, the signs of eosinophilia, and stomach and duodenal ulcers (using gastroscopic endoscope) were considered, as well as the collection of larvae in the stool and lesions of worms under the skin.

### Specific identification of the causal agent

Morphological identification of the nematode worms obtained from the patients was made using the reference guide of Miyazaki [7].

### Molecular characterization of the parasite

For this purpose, a 501-bp sequence of the first internal transcribed spacer ITS1 of the nuclear ribosomal DNA from the collected worms was obtained and analyzed. Indeed, the two spacers ITS1 and ITS2 are known as the best markers for the identification of species in invertebrates in general [38]. Larvae sequenced were from 42 patients from different geographical origins in the zone studied.

For DNA isolation performance, Qiagen kits (DNeasy Blood & Tissue Kit; Qiagen, Gremantown, MD, USA) were used for the extraction of total DNA from *Strongyloides stercoralis* in accordance with the manufacturer’s protocol. In brief, worms were resuspended in the 100 µl of the manufacturer’s lysis buffer ATL (> 8 mM EDTA, > 0.5% SDS) containing 20 µl of proteinase K and incubated at 56 °C for 30 min. Thereafter, 4 µl of RNase and 200 µl of ATL buffer were added and treated in accordance with the manufacturer’s protocol (for a microfuge scale preparation).

PCR for amplification of the 501-bp fragment of the ITS-1 was performed in a 50 μl volume. PCR reactions were performed in 10 mM Tris-HCl, pH 8.4, 50 mM KCl; 3.0 mM MgCl_2_ 250 µM each of dATP, dCTP, dGTP and dTTP; 50 pmol of each primer with 1 U Taq polymerase (Promega, Madison, USA). The following primers were used in separate reaction mixes: S-F (5′-ATC CTT CCA ATC GCT GTT GT-3′) and S-R (5′-TTT CGT GAT GGG CTA ATT CC-3′); Nested-F (5′-GTA ACA AGG TTT TCG TAG GTG AA-3′) and Nested-R (5′-ATT TAG TTT CTT TTC CTC CGC TT-3′) [39]. Amplification was first conducted for 25 cycles using the primer set S-F and F-R. Then, 2 µl of each F-S and F-R amplicon was transferred to a fresh tube containing the same PCR reaction buffer with the primer set Nested-F and F-S and another with the primer set Nested-R and F-R, and amplified for another 35 cycles. Cycling was performed in a Genius Thermal Cycler (Techne, Essex, UK) using the following parameters: initial denaturation at 94 °C for 5 min, followed by 25 cycles (35 cycles in the second PCR) of 94 °C for 30 s (denaturation), 55 °C for 30 s (annealing) and 72 °C for 30 s (extension), followed by a final extension step at 72 °C for 5 min.

Dideoxy sequencing was performed using BigDye™ Terminator Chemistry v.3.1 (Applied Biosystems, Foster City, CA, USA) according to the manufacturer’s instructions. Forward and reverse primers for *Strongyloides stercoralis* were used as sequencing primers using the ABI 3130 Bioanalyzer (Applied Biosystems).

Multiple sequence alignments were performed by using the ATGC software v.7.0.2 and the Clustal W program to determine nucleotide sequence similarities. Phylogenetic trees were constructed in MEGA using the neighbour-joining (NJ) cluster algorithm with evolutionary distances estimated using the Kimura 2-parameter model; bootstrapping was performed using 1000 pseudoreplicates.

## Results

### Epidemiological aspects

From 2000 adult patients comprising 1022 females and 978 males who visited Hanoi Medical University Hospital, a total of 400 patients tested positive by the ELISA test of *S. stercoralis* antigen, furnishing a seroprevalence of *S. stercoralis* of 20%. All larvae-positive individuals proved to be seropositive. Male patients were more frequently seroreactive than females (214/1022, 20.94%; and 186/978, 19.02%, respectively, but the difference was not significant; *P *> 0.05). The prevalence of strongyloidiasis was higher in the older age groups than in the younger age groups (Table 1).

**Table 1:** Seroprevalence and symptoms of strongyloidiasis according to age groups

	Age groups (years)^a^					
	16–25	26–35	36–45	46–55	56–65	> 65
Seroprevalence	19.46 (58/298)	19.02 (70/268)	19.27 (69/358)	20.50 (68/322)	20.45 (73/357)	21.55 (64/297)
Diarrhoea	53.45 (31/58)	60.00 (42/70)	65.22 (45/69)	65.15 (43/66)	67.12 (49/73)	75.00 (48/64)
Digestive disorder	51.72 (30/58)	52.85 (37/70)	56.52 (39/69)	62.12 (41/66)	58.90 (43/73)	65.63 (42/64)
Stomachache	43.10 (25/58)	41.42 (29/70)	44.93 (31/69)	45.45 (30/66)	47.95 (35/73)	50.00 (32/64)
Stomach and duodenal ulcers	39.66 (24/58)	42.85 (30/70)	44.93 (30/69)	43.94 (28/66)	45.21 (32/73)	51.56 (33/64)
Itching	24.14 (14/58)	24.29 (17/70)	24.64 (17/69)	28.79 (19/66)	31.51 (25/73)	34.38 (22/64)
Fever	5.17 (2/58)	7.14 (5/70)	10.14 (7/69)	9.10 (6/66)	10.96 (8/73)	14.06 (9/64)
Bloody stool	3.45 (2/58)	5.71 (4/70)	8.70 (6/69)	9.10 (6/66)	9.59 (7/73)	10.94 (7/64)

^a^Data are given as prevalence (in %) (no. of positive patients/no. of examined patients)

*Strongyloides stercoralis* infection also showed to be higher in rural areas than in urban areas (311/1390 = 22.37% and 89/610 = 14.59%; *P *< 0.05), both in general and individually in all provinces without exception (Table 2). Interestingly, however, seroprevalences proved to be lower in the mountainous areas than in the large valley lowlands (Fig. 1c).

**Table 2:** Geographical distribution of strongyloidiasis in the urban areas and the rural areas of northern Vietnam according to province

Code	Province	Urban areas		Rural areas		Total	
		*n*/*N*	%	*n*/*N*	%	*n*/*N*	%
1	Lai Chau	2/16	12.50	8/37	21.62	10/53	18.87
2	Lao Cai	2/19	10.53	10/51	19.61	12/70	17.14
3	Ha Giang	2/18	11.11	11/49	22.45	13/67	19.40
4	Cao Bang	2/15	13.33	10/48	20.83	12/63	19.48
5	Bac Kan	2/16	12.50	10/51	19.61	12/67	17.91
6	Tuyen Quang	4/27	14.81	12/55	21.82	16/82	19.51
7	Yen Bai	3/22	13.64	11/52	21.15	14/74	18.92
8	Son La	3/21	14.29	11/51	21.57	14/72	19.44
9	Phu Tho	4/28	14.29	12/52	23.08	16/80	20.00
10	Vinh Phuc	2/19	10.52	9/45	20.00	11/64	17.19
11	Thai Nguyen	2/18	11.11	10/47	21.28	12/65	18.46
12	Lang Son	2/17	11.76	11/48	22.92	13/65	20.00
13	Bac Giang	4/21	19.05	12/49	24.49	16/70	22.86
14	Quang Ninh	3/19	15.79	11/53	20.75	14/72	19.44
15	Hai Duong	4/24	16.67	13/55	23.64	16/79	20.25
16	Ha Tay	4/25	16.00	13/56	23.21	18/81	22.22
17	Hoa Binh	4/21	19.05	13/54	24.07	17/75	22.67
18	Thanh Hoa	4/25	16.00	13/54	24.07	17/79	21.52
19	Nghe An	4/24	16.67	12/53	22.64	16/77	20.78
20	Bac Ninh	4/23	17.39	11/52	21.15	15/75	20.00
21	Hai Phong	4/24	16.67	12/52	23.08	16/76	21.05
22	Ha Noi	6/35	17.14	14/58	24.14	20/93	21.51
23	Hung Yen	3/22	13.64	11/49	22.45	14/71	19.72
24	Ha Nam	3/21	14.29	13/56	23.21	16/77	20.78
25	Nam Dinh	5/36	13.89	15/61	24.59	20/97	20.62
26	Thai Binh	5/35	14.29	14/60	23.33	19/95	20.00
27	Ninh Binh	2/19	10.53	9/42	21.43	11/61	18.03
	Whole northern part of Vietnam	89/610	14.59	311/1390	22.37	400/2000	20.00

For the geographical distribution of the provinces see Fig. 1c

### Analysis of symptoms and signs

Of the 400 strongyloidiasis-seropositive patients, results showed that the main symptoms included diarrhoea 64.5% (258/400), digestive disorder 58.0% (232/400), stomachache 45.5% (182/400), itching 28.0% (112/400), fever 9.5% (38/400) and bloody stool 8.0% (32/400). Signs included eosinophilia 100% (400/400) and stomach and duodenal ulcers 44.5% (178/400). The symptoms and signs described were not seen in seronegative and larvae-negative patients. There was no difference in symptoms and signs between larvae-positive and larvae-negative individuals.

**Fig. 2:**
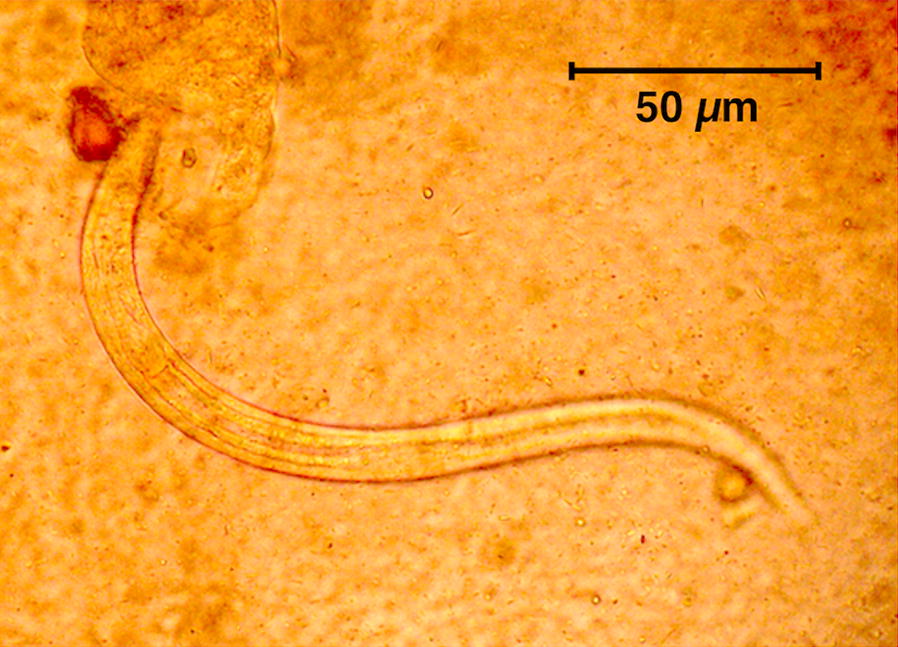
Rhabditiform larva collected from stools of an infected patient from northern Vietnam. Note the rhabditoid oesophagus on the left

**Fig. 3:**
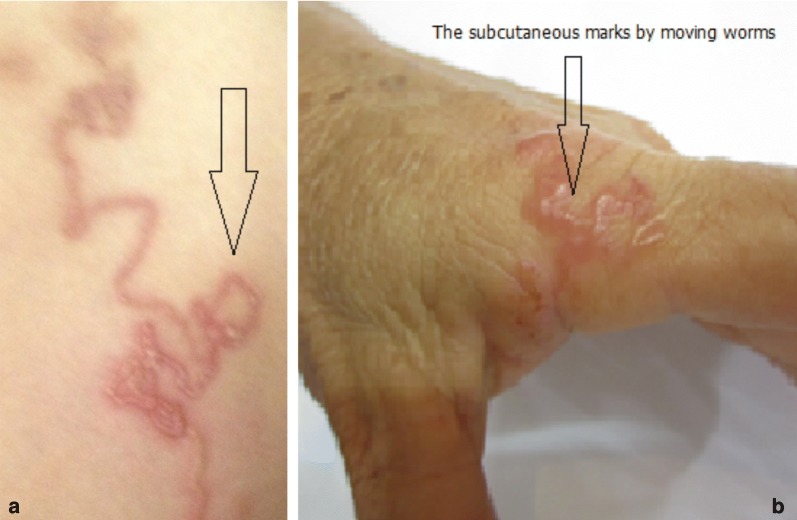
Skin lesions found in Vietnamese patients showing two creeping eruptions (**a**, **b**) of the type of cutaneous larva migrans

Nematode rhabditiform larvae of *S. stercoralis* (Fig. 2) were collected from stools in 10.5% of the patients (42/400), namely in 33 among 328 (10.06%) in the 16–60 years-old group and 9 out of 72 (12.50%) in the > 60 years-old group. Skin lesions showing a creeping eruption of the type of cutaneous larva migrans (Fig. 3) were detected in 3.5% of the patients (14/400).

**Fig. 4:**
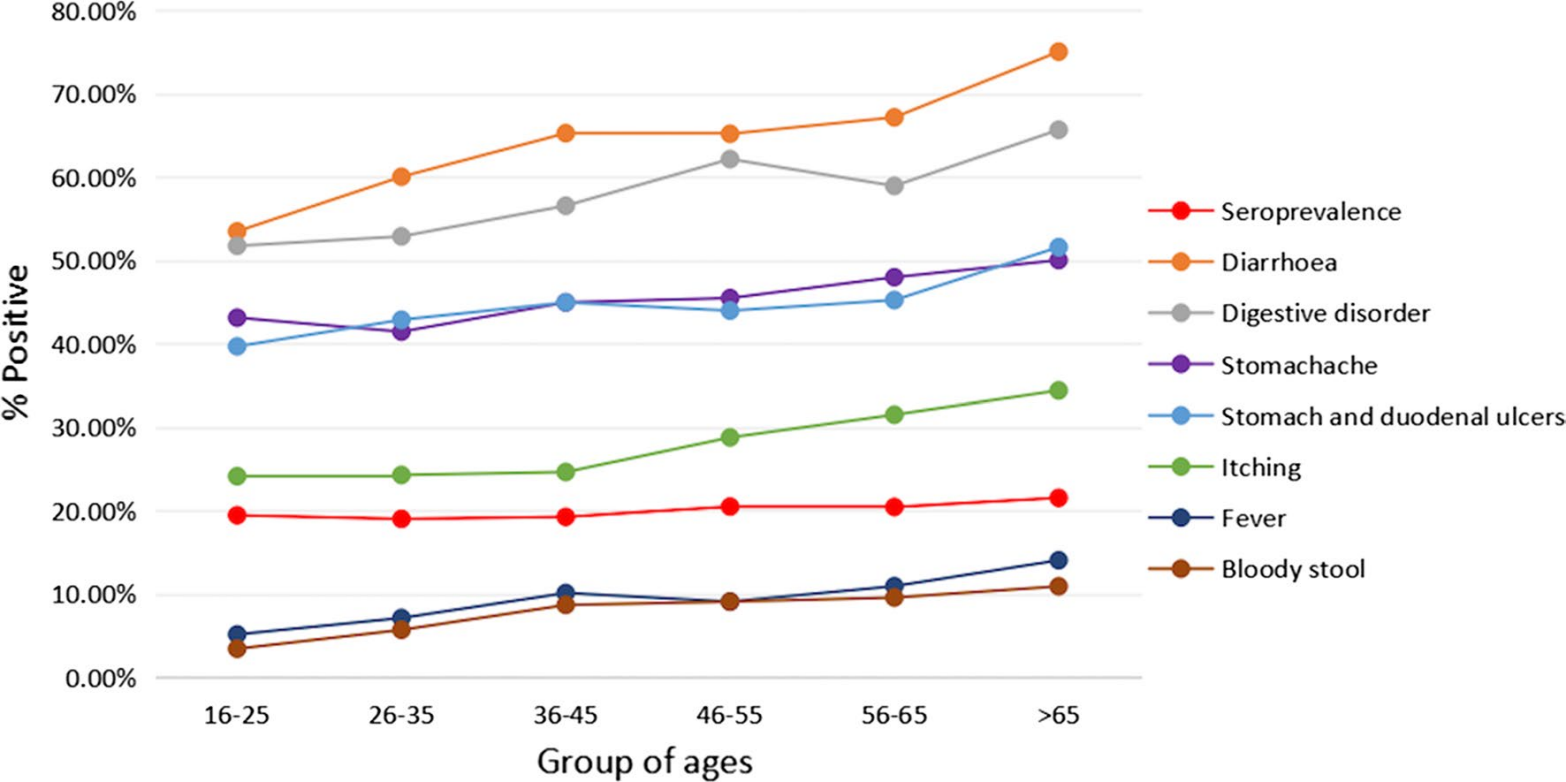
Seroprevalence and symptoms found in strongyloidiasis patients from northern Vietnam related to the age group

When analyzed regarding age, the different groups showed slowly gradually increasing curves from the younger to the older groups (Table 1, Fig. 4). Moreover, these symptoms were more frequently detected in patients from the rural areas than in those from the urban areas.

### DNA sequencing

**Fig. 5:**
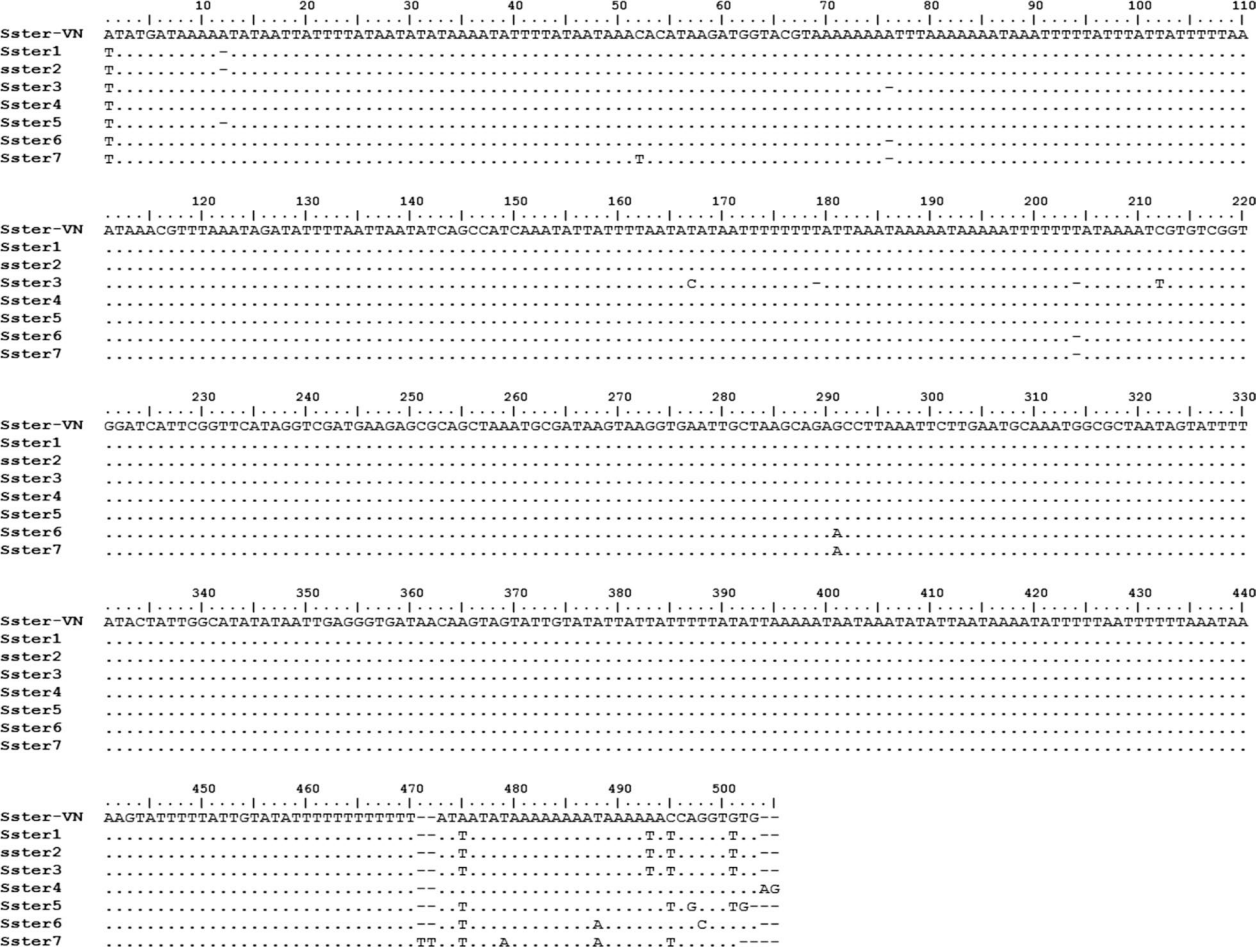
Alignment of ITS1 sequences, including *Strongyloides stercoralis* from Vietnam (Sster-VN) and from other countries (Sster1 to Sster7). (.) = same nucleotide as in the first sequence

**Fig. 6:**
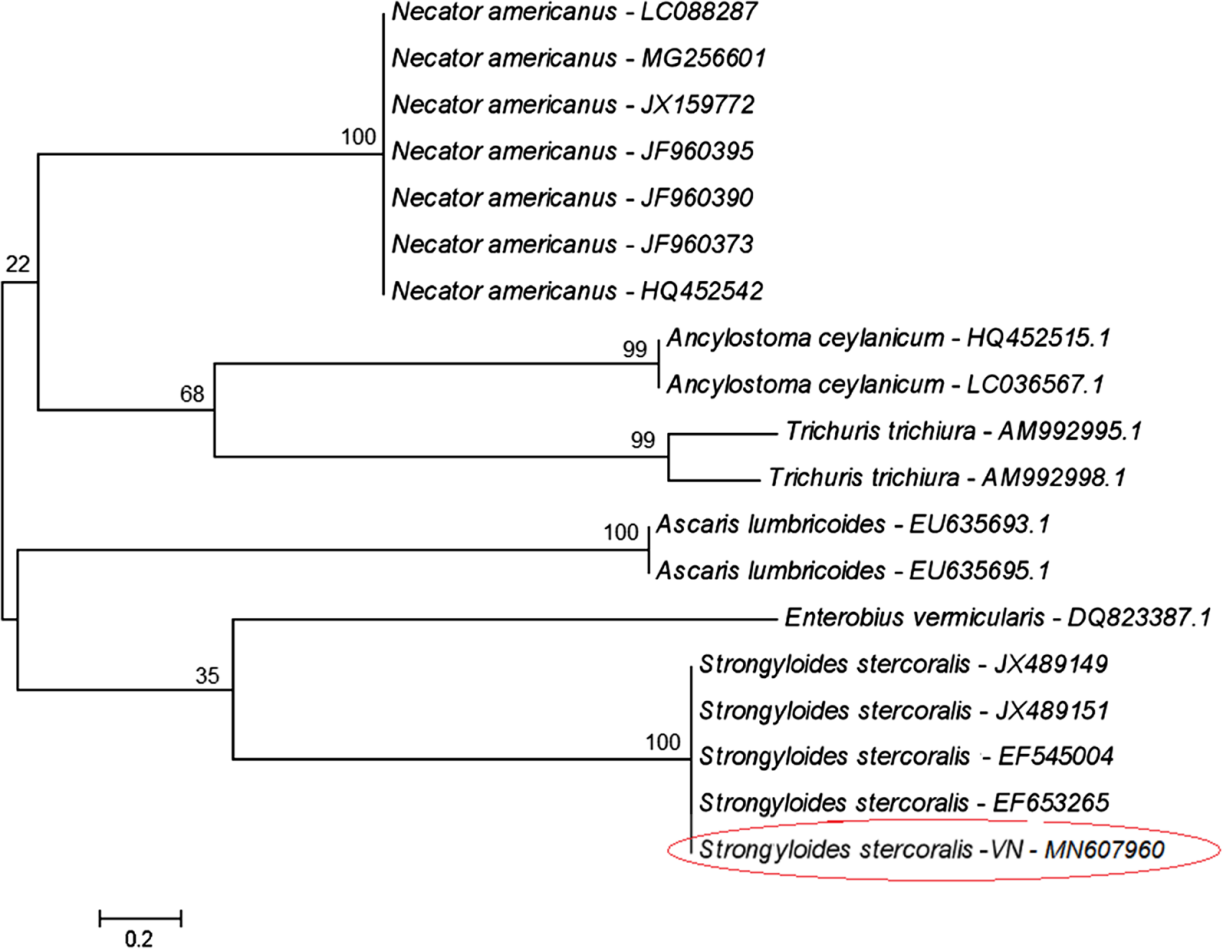
Phylogenetic tree based on ITS1 sequence data including *Strongyloides stercoralis* from Vietnam (VN) and from other countries, together with other human-infecting nematode species available on GenBank

Nematode larvae obtained from patient’s stools were used for the molecular identification and characterization of the causal agent. A 501-nucleotide fragment of the ITS1 of *S. stercoralis* from Vietnam was sequenced and compared with different *S. stercoralis* sequences from GenBank, including Iranian and Australian *S. stercoralis* [40, 41] (Table 3). The results showed a high nucleotide similarity between Vietnamese *S. stercoralis* and other sequences of this species on GenBank at a level of 99% (Fig. 5). In the alignment, only four positions showing transitions and another four positions showing insertions/deletions (indels) appeared scattered throughout most of the sequence length. The majority of the nucleotide differences appeared concentrated in the final part of the sequence, from position 471, including mostly transversions and very scarce indels and transitions throughout 13 varying positions. In the phylogenetic tree including other human-infecting nematodes, Vietnamese *S. stercoralis* clustered in one group together with *S. stercoralis* sequences from GenBank with a 100% support value (Fig. 6).

**Table 3:** Sequences of the internal transcribed spacer 1 (ITS1) of *Strongyloides stercoralis* from Vietnam and other countries

Notation	Origin	Host	Length (bp)	GenBank ID	Reference
Sster-VN	Vietnam	Human	501	MN607960	This study
Sster1	Iran	–	501	EF545004.1	Moghaddassani et al. [40]
Sster2	Iran	–	501	EF653265.1	Moghaddassan et al. [40]
Sster3	Australia	–	501	JX489140.1	Sultana et al. [41]
Sster4	Australia	–	501	JX489145.1	Sultana et al. [41]
Sster5	Iran	–	501	EF653266.1	Moghaddassan et al. [40]
Sster6	Australia	–	501	JX489151.1	Sultana et al. [41]
Sster7	Australia	–	501	JX489149.1	Sultana et al. [41]

## Discussion

The seroprevalence found among a total of 2000 patients from all 27 provinces of northern Vietnam (Fig. 1b, c) who visited Hanoi Medical University Hospital, including an infection prevalence higher in the rural areas than in the urban areas (Table 2), may be considered as relatively high, although it agrees well with seroprevalence data reported previously from this country. In a study of sera selected from a serum bank, the seroprevalence showed to be highest in the rural central highlands (42.4% out of 335 adult subjects from Dak Lak), followed by the central coast area (29.9% of 335 adult subjects from Hue), and with lower prevalences in the big cities such as in Ho Chi Minh (18.2% of 335 adult subjects) and Hanoi (26.0% of 335 adult subjects) [35]. Our study of a larger number of subjects revealed that in the Hanoi capital area strongyloidiasis prevalence is somewhat lower, i.e. 20%, although this is still a worrying public health scenario given the morbidity and potential mortality of this nematode disease. The statistically significant rural/urban relationship of 22.37%/14.59% found in our patients agrees with the previous analyses [35].

**Fig. 7:**
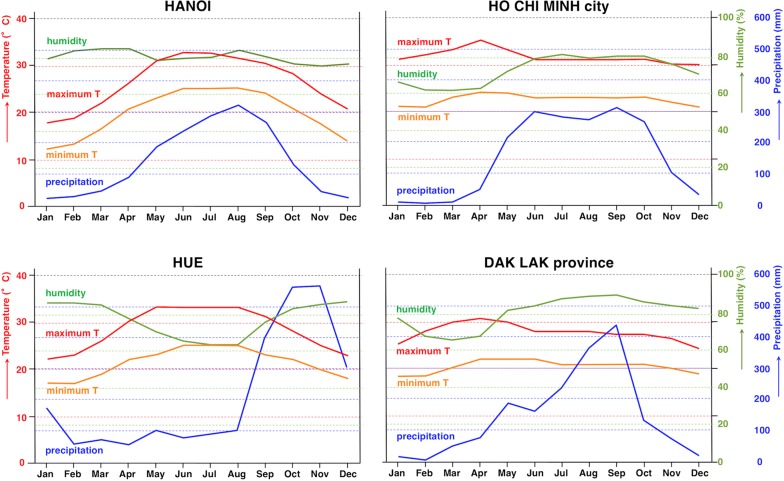
Mean monthly values of climate factors influencing the external cycle of *Strongyloides stercoralis* in (i) Hanoi city representing the northern Vietnam area studied and where the Medical University Hospital receiving the patients studied is located; (ii) Ho Chi Minh city in southern Vietnam lowland; (iii) Hue in the central coast area; and (iv) Dak Lak province in rural south-central highlands

The overall geographical distribution of human strongyloidiasis in Vietnam fits the appropriate climate factor values of maximum temperature, minimum temperature, humidity and rainfall throughout the country (Fig. 7). The aforementioned seroprevalences found by other authors [35] and those found in the present study in northern Vietnam correspond well with the warm temperatures and very high humidity, when taking into account geographical characteristics (north or south location, inland highland or coastal lowland) and human settlement types (urban or rural). Regarding the environmental characteristics which may be considered from the point of view of disease transmission, i.e. the free-living stages of *S. stercoralis* in the soil (adults, larvae), the differences of temperatures throughout the year between the north (represented by Hanoi) and the south (represented by Ho Chi Minh and Dak Lak) concerning seasonality should be highlighted. Thus, whereas in Hanoi temperatures decrease pronouncedly during the winter months (November to February), in Ho Chi Minh and Dak Lak temperatures appear to be stably high the year long (Fig. 7). Given that the duration of time in the environment of the external cycle of *S. stercoralis* is limited to three weeks maximum in an optimum environment, a temperature of 20–28 °C [5], this indicates that in northern latitudes of Vietnam the external cycle may be highly restricted or stopped during winter, whereas on the contrary it may continuously give rise to subsequent generations in southern latitudes. This may explain the higher prevalences in the south when compared to the north.

The aspect of altitude influence in northern Vietnam also merits a comment. Seroprevalences in mountainous areas showed to be slightly lower than in the lowland plains of the wide valley (Fig. 1c). This may be most probably related to the higher human densities facilitating human infection in the lowlands.

In our results, the prevalence in males was only slightly higher than in females, the difference lacking statistical significance. This result does not agree with previous surveys in this country in which a significantly higher prevalence was found in males [35].

The very low number of patients, in whom *Strongyloides* larvae were found in stools, highlights the low sensitivity of the coprological diagnosis of this disease by larvae detection, in agreement with previous studies [25–27].

The distribution of prevalences and symptoms according to age allows for interesting conclusions. Seroprevalences, diarrhoea, digestive disorders, stomachache, stomach and duodenal ulcers, itching, fever and bloody stool (Table 1, Fig. 4) showed slowly increasing curves with increasing age groups which suggests: (i) infection may most probably occur during early child period; (ii) accumulative individual infections in people occurs during life; and (iii) long-term chronicity may be common in infected subjects, mainly in the older age groups. In its turn, this suggests that subjects infected by *Strongyloides* may not go to the hospital for diagnosis and treatment for a long time, until the symtomatology becomes sufficiently worrying. These suggestions are supported by the following considerations: (i) it is well known that *S. stercoralis* can lead to a long-lasting infection; (ii) most of the *S. stercoralis* infections are asymptomatic, so symptoms will not be a key factor for asking for treatment, mainly in children; (iii) the patients examined were not following current and previous or past anti-*Strongyloides* treatments; (iv) there are insufficient conditions of hygiene and sanitation in the rural and also the poor urban areas from where the patients live, and (iv) aged people in those areas mainly depend on agricultural activities in the field.

From the point of view of disease epidemiology, the problem becomes evident if outdoor defecation is practised by these infected subjects, thus assuring the transmission of strongyloidiasis in the area. This will also be the case if the sewage systems cause human stools to be deposited into areas where humans may put bare parts of their skin into direct contact with the contaminated soil, which may take place in Hanoi city surrounding suburbs and rural areas. In that sense, the relatively high proportion of patients (3.5%, 14/400) showing creeping eruption skin lesions should be highlighted. Indeed, these lesions fit typical cutaneous larva migrans infections by animal hookworms, thus referring to the same soil infection source by direct skin contact. Cutaneous larva migrans syndromes are caused by many different species of hookworms of dogs and cats such as most frequently *Ancylostoma brasiliense*, but also secondarily *A. caninum*, *A. ceylanicum*, and rarely *Uncinaria stenocephala*. The serpiginous cutaneous lesions found in the patients suggest that *A. brasiliense* or *A. ceylanicum* were involved, according to the characteristics of the skin lesions which appear to be clearly different from those caused by *A. caninum* [42]. The causal agent of the creeping eruption lesions in our patients should be ascribed to *A. ceylanicum*, taking into account the recent molecular re-evaluation of dog ancylostomids in Vietnam which demonstrated that this is the dog hookworm species present in this country instead of the previously recorded *A. brasiliense* [43].

Interestingly, cutaneous larva migrans lesions were also found in the posterior limbs in 4% of 50 strongyloidiasis-seropositive patients in another study of communities in Vietnam [36]. Symptoms related to *Strongyloides* infection in this study included abdominal pain in 88% of the patients, irregular and intermittent loose stool in 46%, swelling and urticaria in 74%, headache in 78% and weight loss in 12%.

The analysis of the 501-bp nucleotide sequence of the ITS1 rDNA by comparison with the same sequence marker of the species *S. stercoralis* from other countries revealed very few nucleotide differences. When considering the value of the ITS1 as a marker of species [38], it may be concluded that the Vietnamese nematode belongs to the species *S. stercoralis*.

To our knowledge, this is the first molecular identification of *S. stercoralis* in Vietnam. To date, this marker has been obtained from *S. stercoralis* only in Australia [41], Iran [44] and Indonesia [45]. Other DNA markers have more frequently been used for the molecular diagnosis of *S. stercoralis* infection, such as the small subunit *18S* rRNA gene [46–48] and mitochondrial DNA *cox*1 gene [48, 49]. The *18S* gene is a highly conserved sequence [38] which may be used to differentiate other nematode or helminths infecting humans (as clearly illustrated by our phylogenetic tree in which very low bootstrap values appear in the main nodes; Fig. 6), whereas the *cox*1 gene is a more variable marker which may be more useful to analyze intraspecific or inter-population variability [38]. Thus, ITS1 becomes more appropriate for interspecific differentiation, which in the case of *Strongyloides* spp. infection in humans becomes important due to the existence of another *Strongyloides* species able to infect humans, *S. fuelleborni* [45, 47, 50] or even non-*Strongyloides* rhabditids which may appear in human stools [51].

## Conclusions

To our knowledge, this study is the largest survey of human strongyloidiasis in Vietnam to date and the first molecular identification of *S. stercoralis* in the country. Further molecular studies of *S. stercoralis*-infected patients from throughout Vietnam with the same DNA marker are needed to assess the potential intraspecific variability and thus enable conclusions about the zoonotic origin of human infections. This may help in designing appropriate control measures at the level of animal reservoirs and transmission pathways. The higher seroprevalences in rural areas than in urban areas, both in general and individually in all provinces without exception, indicates the convenience to concentrate epidemiological efforts in rural areas not yet surveyed in Vietnam. The higher prevalence of 44.7% recently found by coprological methods in rural Cambodia [52] suggests that a higher public health problem by *S. stercoralis* may be present in more remote areas. Long-term chronicity may be common in infected subjects, mainly in the older age groups. The stable high temperatures in southern Vietnam and seasonal temperatures including cooler winters in the northern Vietnam may explain the lower prevalences in the north.

## Data Availability

Data supporting the conclusions of this article are included within the article. The newly generated sequence was submitted to the GenBank database under the accession number MN607960.

## Abbreviations

ELISA: enzyme-linked immunosorbent assay
ITS1: internal transcribed spacer 1
